# Identification of ciliogenic compounds using pancreatic cancer cells as a screening model

**DOI:** 10.1186/2046-2530-4-S1-P57

**Published:** 2015-07-13

**Authors:** N Khan, N Willemarck, J Swinnen

**Affiliations:** 1 Dept. of Oncology, Lab. of Lipid Metabolism in Cancer, KU Leuven, Leuven, Belgium

## Objective

Primary cilia regulate intracellular signaling pathways by sensing the extracellular environment. Abnormalities in the structure and/or function of cilia results in deregulation of these pathways leading to clinically significant diseases like cancer. In the present study, we demonstrate the use of cancer cells in the development of a cell culture-based semi-high throughput screening method to identify ciliogenic compounds that may serve as potential anti-cancer agents. Using this method, we have identified compounds that enhance ciliation in pancreatic cancer cells.

## Methods

A screening platform was developed by culturing cancer cells in a 96-well plates followed by immunostaining for cilia. Quantification of cilia was accomplished by analyzing images captured by INCell Analyzer, an automated machine used for image acquisition. Ciliogenic compounds were identified based on percentage of ciliated cells. The hits were further assessed in cancer cells. Their ability to decrease the proliferation of cancer cells was analyzed by spheroid assay. Western Blots were performed to identify the pathways through which ciliogenesis was induced by these compounds.

## Results

26 such compounds were identified which enhanced ciliation in pancreatic cancer cells. Based on their targets, they were classified into 14 different classes. Most of these compounds effectively decreased the rate of proliferation in pancreatic cancer cells. Two of these compounds namely Gefitinib and Rapamycin suppressed the expression levels of transcription factor SREBP1c, which is known to be overexpressed in cancer cells.

## Conclusion

Via development of a semi-high throughput screening platform, 26 ciliogenic compounds were identified which may serve as anti-cancer agents by virtue of their ability to induce cilia in cancer cells.

